# Synonymous *GATA2* mutations result in selective loss of mutated RNA and are common in patients with GATA2 deficiency

**DOI:** 10.1038/s41375-020-0899-5

**Published:** 2020-06-18

**Authors:** Emilia J. Kozyra, Victor B. Pastor, Stylianos Lefkopoulos, Sushree S. Sahoo, Hauke Busch, Rebecca K. Voss, Miriam Erlacher, Dirk Lebrecht, Enikoe A. Szvetnik, Shinsuke Hirabayashi, Ramunė Pasaulienė, Lucia Pedace, Marco Tartaglia, Christian Klemann, Patrick Metzger, Melanie Boerries, Albert Catala, Henrik Hasle, Valerie de Haas, Krisztián Kállay, Riccardo Masetti, Barbara De Moerloose, Michael Dworzak, Markus Schmugge, Owen Smith, Jan Starý, Ester Mejstrikova, Marek Ussowicz, Emma Morris, Preeti Singh, Matthew Collin, Marta Derecka, Gudrun Göhring, Christian Flotho, Brigitte Strahm, Franco Locatelli, Charlotte M. Niemeyer, Eirini Trompouki, Marcin W. Wlodarski, European Working Group of MDS in Childhood (EWOG-MDS)

**Affiliations:** 1 Division of Pediatric Hematology and Oncology, Department of Pediatrics and Adolescent Medicine, Medical Center, Faculty of Medicine, University of Freiburg, Freiburg, Germany; 2 Faculty of Biology, University of Freiburg, Schänzlestraße 1, 79104 Freiburg, Germany; 3 Department of Cellular and Molecular Immunology, Max Planck Institute of Immunobiology and Epigenetics, Freiburg, Germany; 4 Department of Hematology, St. Jude Children´s Research Hospital, Memphis, USA; 5 Institute of Molecular Medicine and Cell Research, University of Freiburg, Freiburg, Germany; 6 Lübeck Institute of Experimental Dermatology and Institute of Cardiogenetics, University of Lübeck, Lübeck, Germany; 7 Comprehensive Cancer Center Freiburg (CCCF), University Medical Center, Faculty of Medicine, University of Freiburg, Freiburg, Germany; 8 German Cancer Consortium (DKTK), Freiburg, Germany; 9 German Cancer Research Center (DKFZ), Heidelberg, Germany; 10 Department of Pediatrics, Hokkaido University Graduate School of Medicine, Sapporo, Japan; 11 Vilnius University Hospital Santaros Klinikos, Center for Pediatric Oncology and Hematology, Bone Marrow Transplantations Unit, Vilnius, Lithuania; 12 Department of Pediatric Hematology and Oncology, Istituto di Ricovero e Cura a Carattere Scientifico Ospedale Pediatrico Bambino Gesù, Rome, Italy; 13 Genetics and Rare Diseases Research Division, Istituto di Ricovero e Cura a Carattere Scientifico Ospedale Pediatrico Bambino Gesù, Rome, Italy; 14 Department of Pediatric Pneumology, Allergy and Neonatology, Hannover Medical School, Hannover, Germany; 15 Institute of Medical Bioinformatics and Systems Medicine, Medical Center—University of Freiburg, Faculty of Medicine, University of Freiburg, Freiburg, Germany; 16 Department of Hematology and Oncology, Hospital Sant Joan de Déu, Barcelona, Spain; 17 Department of Pediatrics, Aarhus University Hospital Skejby, Aarhus, Denmark; 18 Dutch Childhood Oncology Group (DCOG), Princess Máxima Centre, Utrecht, The Netherlands; 19 Central Hospital of Southern Pest—National Institute of Hematology and Infectious Diseases, Budapest, Hungary; 20 Department of Pediatric Oncology and Hematology, University of Bologna, Bologna, Italy; 21 Department of Pediatric Hematology-Oncology and Stem Cell Transplantation, Ghent University Hospital, Ghent, Belgium; 22 St. Anna Children´s Hospital and Cancer Research Institute, Pediatric Clinic, Medical University of Vienna, Vienna, Austria; 23 Department of Hematology and Oncology, University Children’s Hospital, Zurich, Switzerland; 24 Paediatric Oncology and Haematology, Our Lady’s Children’s Hospital Crumlin, Dublin, Ireland; 25 Department of Pediatric Hematology and Oncology, Charles University and University Hospital Motol, Prague, Czech Republic; 26 Department of Paediatric Bone Marrow Transplantation, Oncology and Hematology, Medical University of Wroclaw, Wroclaw, Poland; 27 Institute of Immunity and Transplantation, University College London (UCL), London, UK; 28 Bone Marrow Transplant (BMT) Programme, UCL Hospital National Health Service Foundation Trust (NHS FT), London, UK; 29 Department of Immunology, Royal Free London NHS FT, London, UK; 30 Institute of Cellular Medicine, Newcastle University, Newcastle upon Tyne, UK; 31 NIHR Newcastle Biomedical Research Centre at Newcastle upon Tyne Hospitals NHS Foundation Trust, Newcastle upon Tyne, UK; 32 Institute of Human Genetics, Hannover Medical School, Hannover, Germany; 33 Department of Pediatrics, Sapienza University of Rome, Rome, Italy; 34 CIBSS—Centre for Integrative Biological Signaling Studies, Freiburg, Germany

**Keywords:** Haematological diseases, Genetics research

## Abstract

Deficiency of the transcription factor GATA2 is a highly penetrant genetic disorder predisposing to myelodysplastic syndromes (MDS) and immunodeficiency. It has been recognized as the most common cause underlying primary MDS in children. Triggered by the discovery of a recurrent synonymous *GATA2* variant, we systematically investigated 911 patients with phenotype of pediatric MDS or cellular deficiencies for the presence of synonymous alterations in *GATA2*. In total, we identified nine individuals with five heterozygous synonymous mutations: c.351C>G, p.T117T (*N* = 4); c.649C>T, p.L217L; c.981G>A, p.G327G; c.1023C>T, p.A341A; and c.1416G>A, p.P472P (*N* = 2). They accounted for 8.2% (9/110) of cases with GATA2 deficiency in our cohort and resulted in selective loss of mutant RNA. While for the hotspot mutation (c.351C>G) a splicing error leading to RNA and protein reduction was identified, severe, likely late stage RNA loss without splicing disruption was found for other mutations. Finally, the synonymous mutations did not alter protein function or stability. In summary, synonymous *GATA2* substitutions are a new common cause of GATA2 deficiency. These findings have broad implications for genetic counseling and pathogenic variant discovery in Mendelian disorders.

## Introduction

Germline mutations in the *GATA2* gene, mostly arising de novo, had been reported to cause an immunodeficiency/myelodysplasia syndrome manifesting with a multitude of clinical phenotypes. These include monocytopenia and mycobacterial infections syndrome (MonoMAC syndrome) [1], dendritic cell, monocyte, B and NK lymphoid deficiency (DCML deficiency) [2], familial myelodysplastic syndrome (MDS)/acute myeloid leukemia (AML) [3], chronic neutropenia [4], Emberger syndrome [5] and warts, immunodeficiency, lymphedema and anogenital dysplasia syndrome (WILD syndrome) [6]. Finally, GATA2 deficiency is considered the most common hereditary predisposition to pediatric MDS, accounting for as much as 15% of MDS with excess of blasts (MDS-EB), with a particularly high prevalence among MDS patients carrying monosomy 7 (37%) [7]. To date, more than 400 GATA2-deficient cases have been published [8, 9] with three major types of pathogenic *GATA2* mutations: (1) missense mutations within zinc finger 2 (ZnF2), (2) null mutations (splice site, nonsense, frameshift, and whole gene deletions), and (3) noncoding substitutions in the EBOX-GATA-ETS regulatory region in intron 4 (hg19, g.128202128-128202173, NM_032638.4) [8–10]. Overall, germline *GATA2* mutations are thought to result in haploinsufficiency and context-dependent loss of essential transcription factor activity [3, 5, 11–14].

Genomic studies typically focus on the discovery of nonsynonymous variants that alter coding regions or canonical splice sites because their effect is predictable. Conversely, due to codon degeneracy, synonymous substitutions do not alter the amino acid composition of the encoded protein and are usually not reported as pathogenic. However, previous studies revealed that such variants can alter RNA or protein on multiple levels including pre-mRNA splicing, messenger RNA (mRNA) stability and structure, miRNA binding, and translation [15–24].

Here, we initially identified a synonymous substitution in exon 3 of the *GATA2* gene (c.351C>G, p.T117T) in two unrelated pedigrees, with the clinical phenotype of GATA2 deficiency. The variant was recently reported in an adult patient (the mother of two siblings studied here) presenting with immunodeficiency, severe infections and lung disease [25]. This prompted us to study the contribution of synonymous alterations to the genetic spectrum of GATA2 deficiency and to assess their pathogenic role. We discovered and characterized five distinct synonymous mutations with RNA-deleterious effect in nine patients. They represent a new type of mutation in GATA2 deficiency and have broad implications for both the discovery of disease-causing mutations and genetic counseling.

## Methods

### Patient cohort and genomics

**Fig. 1:**
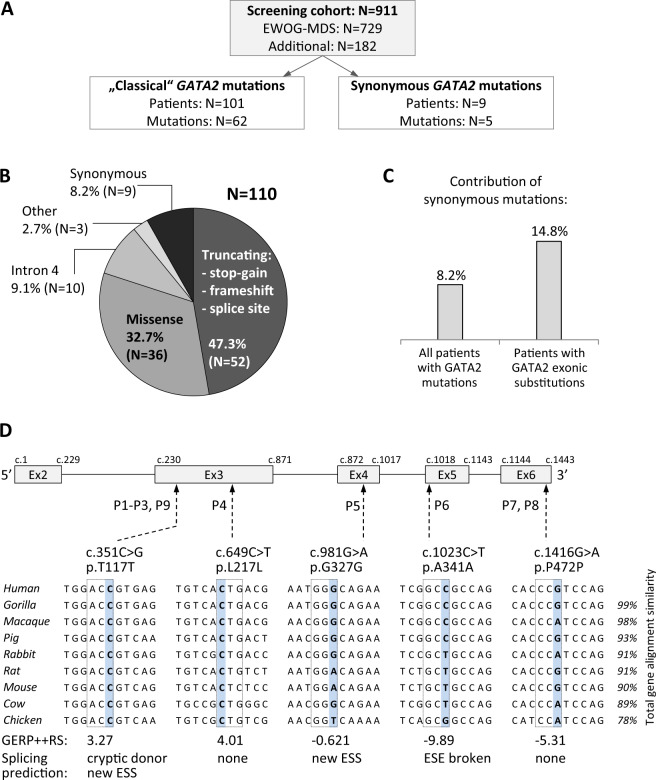
Composition and genetics of the study cohort. **a** Flow diagram depicts the screening cohort and *GATA2* mutations identified. **b** Overall distribution of genotypes among 110 patients with GATA2 deficiency. Truncating variants are localized prior to or within zinc finger 2; missense mutations cluster mainly to zinc finger 2 region; intron 4 mutations affect the EBOX-GATA-ETS regulatory region (+9.5 kb) of *GATA2*; other: one in-frame deletion and two whole gene deletions; synonymous variants are proposed as a new group of pathogenic *GATA2* mutations. Numbers in parentheses refer to individual patients. **c** Frequency of patients with synonymous mutations among all *GATA2* positive cases and among the group of patients carrying exonic substitutions. **d** Schematic representation of the *GATA2* gene (NM_032638.4) with synonymous variants identified. Affected nucleotide is shaded blue and dashed line boxes indicate respective codon triplet. Nucleotide conservation is presented for nine species. Evolutionary conservation is depicted on the bottom as Genomic Evolutionary Rate Profiling (GERP + + RS) score with values ranging from −12.36 to 6.18, and 6.18 being the most conserved. Splicing prediction was performed with Human Splicing Finder v.3.0. ESS exonic splicing silencer, ESE exonic splicing enhancer.

The screening cohort consisted of 911 patients (Fig. 1a): 729 children and adolescents with primary MDS classified according to WHO criteria [26–28] enrolled in the studies 1998 and 2006 of the European Working Group of MDS in Childhood (EWOG-MDS, #NCT00662090), and 182 patients with cytopenias and/or GATA2-specific clinical problems, referred to our diagnostic laboratory. *GATA2* gene sequence, including intron 4 was analyzed in bone marrow (BM) samples using targeted deep sequencing with Sanger sequence validation, and subsequent confirmation of germline mutational status in nonmyeloid tissues as previously reported [7, 29]. Whole exome/genome sequencing (WES/WGS) was performed in patients with synonymous *GATA2* variants to rule out other hereditary causes (Supplementary methods, Supplementary Table 1).

### Targeted investigations of GATA2 transcript expression

We analyzed RNA expression in blood, BM or fibroblasts using Sanger, deep sequencing, and TA cloning-based sequencing (Supplementary methods, Supplementary Fig. 1 and Supplementary Table 2). In addition, *GATA2* expression in various hematopoietic compartments of healthy controls was measured (Supplementary methods).

### Studies of GATA2 protein stability and function

In order to explore the influence of synonymous mutations on protein stability and function, in vitro analysis of exogenously expressed *GATA2* was performed in 293T cells. To further investigate the protein function, in vivo studies in zebrafish were accomplished (for details see Supplementary methods). Experiments were performed in duplicates or triplicates as indicated in the figure legends.

### Statistics

For reporter assay, data from biological and technical triplicate experiments were presented as the mean values ± standard deviation (SD). Statistical significance was assessed using GraphPad Prism v 7.04 software employing either standard one-way ANOVA test (reporter assay, thermodynamic effect of GATA2 variants) or Student’s *t* test (allele quantification in patients’ cDNA by deep sequencing, frequency of zebrafish phenotypes). *P* values < 0.05 were considered statistically significant.

## Results

### Identification of synonymous GATA2 variants

We initially discovered two unrelated individuals (P1, P3) with GATA2 deficiency carrying an identical synonymous *GATA2* variant. This prompted a systematic evaluation of the *GATA2* gene sequence in our screening cohort of patients presenting for the most part with the phenotype of pediatric MDS (Fig. 1a). At first, we categorized “classical” disease-causing alterations and identified 101 patients with 62 distinct pathogenic *GATA2* mutations (Fig. 1a). The distribution of mutations corroborated data reported in previous studies [9]. The most common were null mutations affecting the N-terminal part of the protein: stop-gain, frameshift, splice site (*N* = 52), followed by missense mutations within or adjacent to ZnF2 (*N* = 36), intron 4 EBOX-GATA-ETS site alterations (*N* = 10), and other aberrations (*N* = 3): one in-frame and two whole gene deletions (Fig. 1b).

Next, we searched *GATA2* coding sequence for the presence of synonymous substitutions. Variants that are either not reported or very rare (<0.05% allele frequency) in the gnomAD population database were found in nine patients. These variants were present in 8.2% (9/110) of all patients with *GATA2* alterations, and 14.8% (9/61) of cases with *GATA2* exonic substitutions only (Fig. 1c). In comparison, common polymorphisms with synonymous effect p.P5P, p.P22P, p.Q38Q, p.T188T, and p.A411A were not significantly enriched in our cohort (not shown), arguing against their disease-causing role in MDS.

The synonymous substitutions encountered in P1–P9 were predicted to have a likely benign effect using the combined annotation-dependent depletion score (CADD) and gene-specific calibration by Gene-Aware Variant INterpretation (GAVIN) (Table 1). The evolutionary nucleotide conservation was high for c.351 and c.649 nucleotides (Fig. 1d), suggesting their resistance to evolutionary change. Splicing prediction tools assigned a high chance of splice defects to c.351C>G, c.981G>A, and c.1023C>T variants either via activation of a cryptic donor, introduction of an alternative splicing silencer or disruption of an existing splicing enhancer (Fig. 1d, Supplementary Fig. 2, and Table 1).

**Table 1:** Synonymous variants identified in MDS patients.

Patient no. (ID)	*GATA2* mutation	Genomic DNA VAF% (total depth)	cDNA VAF% WT/Mut (total depth)	Evol conser/ PhysChem diff	CADD/GAVIN (C2; *P* > 26, *B* < 19)	gnomAD browser MAF% (mutant/total)	Splicing prediction
P1 (D 1239)	c.351C>G; p.T117T	^WES^50% (160), ^DS^47% (766)	^DS^99.88%/0.006% (320215)	Medium/none	10.5/B	None	Cryptic donor, new ESS site
P2 (sister of P1)	c.351C>G; p.T117T	Heterozygous (Sanger)	Not done	As above	As above	As above	As above
P3 (D 749)	c.351C>G; p.T117T	^WES^47% (130), ^DS^51% (703)	^DS^99.89%/0.004% (838094)	As above	As above	As above	As above
P4 (LT)	c.649C>T; p.L217L	^WES^48% (196), ^DS^51% (725)	^DS^79.43%/20.56% (429616)	High/none	10.7/B	0.001% (2/246096)	None
P5 (D 722)	c.981G>A; p.G327G	^WES^52% (156), ^DS^48% (788)	^DS^99.96%/0.014% (7190)	Medium/none	18.5/B	None	New ESS site
P6 (D 1142)	c.1023C>T; p.A341A	^WES^47% (296), ^DS^49% (1449)	^DS^99.91%/0.095% (4213)	Weak/none	15.4/B	0.002% (6/275438)	ESE site broken
P7 (D)	c.1416G>A; p.P472P	^WES^49% (63)	^DS^99.91%/0.085% (388784)	Medium/none	12.4/B	0.027% (70/256322)	None
P8 (I 386)	c.1416G>A; p.P472P	Heterozygous (Sanger)	Not done	As above	As above	As above	As above
P9 (UKA2604)	c.351C>G; p.T117T	Heterozygous (Sanger)	Not done	Medium/none	10.5/B	None	Cryptic donor, new ESS site

Gene annotation: *GATA2* (NM_032638.4).

*VAF* variant allelic frequency, *WT* wild-type allele, *Mut* mutated allele, *WES* whole exome sequencing, *DS* deep sequencing, *Sanger* identified by Sanger sequencing, *Evol* (evolutionary) conservation assessed using Phylop and PhastCons, *PhysChem diff* physicochemical difference between amino acids, *CADD* combined annotation-dependent depletion score, *GAVIN* Gene-Aware Variant Interpretation (C2: CADD scores significantly predictive for pathogenicity (*p* < 0.05), *P* pathogenic if CADD > 26, *B* benign if CADD lower than 19), *MAF* minor allelic frequency, *Splicing prediction* Human Splicing Finder v. 3.0. *ESS* exonic splicing silencer, *ESE* exonic splicing enhancer.

### Phenotype of patients with synonymous *GATA2* mutations

Patients with synonymous *GATA2* mutations were diagnosed at a median age of 11.5 (3–24) years. Hematologic and immunological phenotypes were consistent with the heterogeneous clinical picture of GATA2 deficiency and included varying degrees of immune cytopenias (low B/NK, DC cells, monocytopenia), immunodeficiency, neutropenia, and/or pancytopenia (supplemental case descriptions). P2 is the sibling of GATA2-deficient patient P1 and was categorized as a silent *GATA2* mutation carrier with a reduction of B- and NK-cells. Their mother was previously reported with pulmonary alveolar proteinosis [25]. P7 and P8 (unrelated, carrying the same mutation), initially presented with thrombocytopenia and while P8 developed transfusion-dependent refractory cytopenia of childhood (RCC), P7 remained stable with BM morphology suspicious for RCC. P9 was first seen with complications of immunodeficiency and clinically evolved to MDS. Monosomy 7 in BM was detected at diagnosis in four patients (P1, P3, P4, and P6), normal karyotypes were present in four (P5, P7, P8, and P9), while no marrow exam was performed in P2 (Table 2). According to the WHO classification, P1 and P4–P8 were diagnosed with RCC, and P9 with MDS with multilineage dysplasia as a young adult. Initial disease of P3 was MDS-EB, which progressed to AML after 6 months. Other clinical problems in the affected patients were transient organ dysfunction after birth and facial abnormalities in P4, hepatosplenomegaly in P5, hypospadias in P6, and Crohn’s colitis as well as HPV-driven neoplasia in P9. The majority of patients (6/9) underwent allogeneic hematopoietic stem cell transplantation (HSCT) with favorable outcome: 5/6 patients were alive at last follow up (at a median of 1.9 years after HSCT) and 1/6 (P3) died from infection 7 months following HSCT (Table 2).

**Table 2:** Clinical characteristics of patients with synonymous *GATA2* mutations.

Patient no. (ID)	Age at Dx	Sex	Hematological presentation and other features	Karyotype	Therapies	Age and status at last FUP
P1 (D 1239)	12	F	RCC, low IgG, low monocytes/B/DC	−7	MUD-HSCT	13.5 years: alive
P2 (sister of P1)	11	F	B/NK-cell lymphopenia, low IgA/G	Not done	Observation	12 years: alive
P3 (D 749)	14	F	MDS-EB	−7	CB-HSCT	15.4 years: died from infection 7 months after HSCT
P4 (LT)	3	M	RCC, facial abnormalities, skin hypopigmentation, joint hypermobility	−7	MSD-HSCT	7.6 years: alive
P5 (D 722)	11	M	RCC, hepatosplenomegaly	Normal	Observation	18.5 years: alive
P6 (D 1142)	11.5	M	RCC, hypospadias	−7	MUD-HSCT	15.2 years: alive
P7 (D)	14	F	Suspicious for RCC	Normal	–	20.9 years: alive
P8 (I 386)	4	F	RCC	Normal	MUD-HSCT	4 years: alive
P9 (UKA2604)	24	F	MDS-MLD, low monocytes/B/NK/DC, recurrent viral warts, mycobacterium avium infections, HPV-driven neoplasia, Crohn’s colitis	Normal	MUD-HSCT	32 years: alive

*Dx* diagnosis, *RCC* refractory cytopenia of childhood, *DC* dendritic cells, *NK* natural killer cells, *MDS-EB* myelodysplastic syndrome with excess blast, *MDS-MLD* myelodysplastic syndrome with multilineage dysplasia, *HSCT* allogeneic hematopoietic stem cell transplantation, *−7* monosomy 7, *MUD* matched unrelated donor, *CB* cord blood, *MSD* matched sibling donor, *FUP* follow-up.

### Exclusion of other hereditary causes

We next aimed to determine if other genetic conditions predisposing to inherited bone marrow failure (IBMF) or MDS might have been previously missed in our patients. WES/WGS was performed in all families with exception of P8 who was assessed by a 135 IBMFS/MDS gene panel. The WES analysis focused on known IBMF/MDS and pancancer genes (300 genes) [26, 30]. Multiple heterozygous variants of uncertain significance (VUS) were identified (Supplementary Table 1). After comprehensive review by a multidisciplinary board representing pediatric hematology, genetic counseling, and molecular biology, only P6 remained with additional potentially pathogenic *SAMD9* variants p.K877E and p.F366LfsX33. This patient did not have features typical for MIRAGE syndrome, which was initially ascribed to *SAMD9* mutations [31, 32]. Notably, we discovered VUS in the Fanconi anemia (FA) genes *FANCD1, FANCD2*, and *FANCS* in three patients. However, these VUS were heterozygous and FA was ruled out in all three patients by means of chromosomal breakage studies and clinical phenotyping.

### Synonymous *GATA2* variants result in selective loss of mRNA expression

**Fig. 2:**
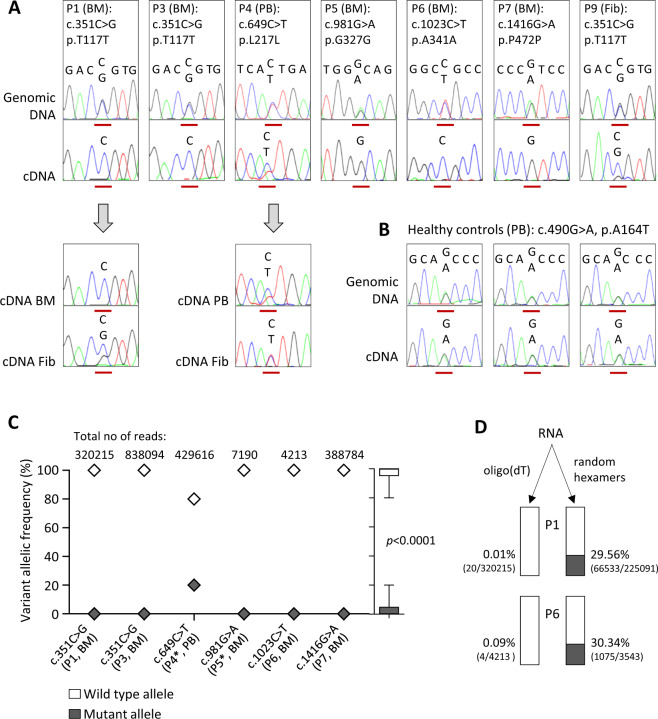
Sequencing analysis of patients with synonymous *GATA2* mutations. **a** Upper panel: Representative electropherograms with genomic and cDNA sequence surrounding the affected nucleotide (red line). All five distinct synonymous mutations are represented. Lower panel: Comparison of allelic expression in the hematopoietic and nonhematopoietic tissue of P1 and P4. **b** Genetic testing of healthy controls carrying a common nonsynonymous *GATA2* polymorphism c.490G>A (19.5% minor allele frequency in gnomAD). **c** Allelic frequency of *GATA2* alleles determined by targeted deep sequencing of patients’ cDNA; numbers of reads taken from one representative replicate. In all cases oligo(dT) priming was used with exception of P4 and P5 (*) where the mixture of random hexamers and oligo(dT) was utilized. Outside right: Boxplot depicts combined allelic contribution in all patients. Calculation of *p* value was performed using Student’s *t* test (mean ± SD values). **d** Frequency of mutated alleles determined by deep sequencing of cDNA obtained from bone marrow RNA using two different reverse transcription priming methods. Mutant vs. total read counts are shown in parentheses, and percentage represents the proportion of mutated alleles in the sample. BM bone marrow, Fib fibroblasts, PB peripheral blood.

Building on the assumption that synonymous variants detected in our patients were associated with degradation of the mutant (Mut) mature mRNA, we first sequenced cDNA transcribed from polyadenylated RNA transcripts (equivalent to mRNA) using Sanger method. Compared with genomic DNA, cDNA sequences showed loss of heterozygosity manifested by complete lack of the Mut allele in five out of seven cases: P1, P3, P5, P6, and P7, and a substantial reduction in P4 and P9 (Fig. 2a upper panel). Compared with hematopoietic specimens, Mut allele expression was slightly higher in skin fibroblasts of P1 and P4 (Fig. 2a lower panel). Because it is not known if monoallelic *GATA2* expression might be a general phenomenon in normal hematopoiesis, we sequenced three healthy controls who carried a common heterozygous polymorphism (rs2335052: c.490G>A; p.A164T). Both the genomic DNA and cDNA showed an equal ratio of alternative to reference alleles (Fig. 2b).

Deep sequencing based quantification of allelic frequency showed nearly total absence of Mut alleles in P1, P3, P5–P7, and a reduction of Mut expression to 21% in P4 (Table 1 and Fig. 2c). Combined across all samples, we observed median values of 27 reads for Mut, versus 330,544 reads for wild-type (WT) alleles. Lastly, TA cloning of P5’s and P6’s cDNA followed by sequencing of an average of 345 single colonies was a third independent method confirming the RNA reduction (0% and 11% of Mut amplicons for P5 and P6, respectively, not shown). In order to address at which stage of RNA maturation the Mut alleles were lost, we deep sequenced products that were reverse-transcribed using alternative priming approaches. While oligo(dT) that are specific to mature transcripts (mRNA) produced almost exclusively *GATA2* WT reads, the use of random hexamers (enriching both pre-mRNA and mRNA) resulted in an increase of Mut reads to ~30% for P1 and P6 (Fig. 2d).

### Splicing analysis of the *GATA2* gene

**Fig. 3:**
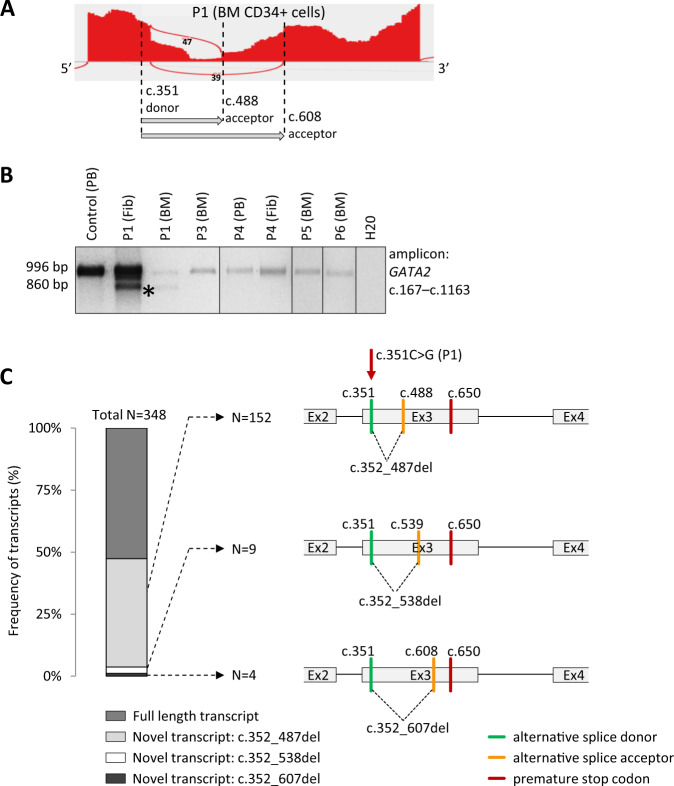
Alternative splicing. **a** Sashimi plot of *GATA2* exon 3 of P1 depicting two novel splicing events (represented by arcs) detected by RNAseq; dashed lines indicate the positions of an alternative donor and two new acceptors. **b** Long range RT-PCR spanning exon 2–5 of *GATA2* transcript revealed the presence of an additional shorter product of 860 bp in P1 (indicated by asterisk (*)) corresponding to one of the transcripts found by RNAseq (donor: c.351—acceptor: c.488). Only wild-type allele (996 bp) was detected in P3–P6. **c** Frequency and schematic representation of novel splicing patterns in P1 detected by TA cloning of the RT-PCR product. It confirmed the presence of three novel transcripts, of which two were also identified by RNAseq. BM bone marrow, Fib fibroblasts, PB peripheral blood. *GATA2* (NM_032638.4).

In order to ascertain the mechanism of monoallelic *GATA2* expression, RNA sequencing (RNAseq) was performed in sorted CD34+ BM cells of five patients (P1, P4–P7). Isoform analysis revealed two novel splice junctions in P1, not observed in the Ensembl database and healthy controls (Fig. 3a and Supplementary Fig. 3). In both new transcripts in P1 the c.351C>G mutation acts as a new splice donor that joins to alternative acceptors either at c.488 or at c.608. Long range RT-PCR and sequencing in P1′ BM and fibroblasts (Fig. 3b) confirmed the presence of the transcript with c.488 alternative acceptor. Finally, TA cloning of the cDNA PCR products of P1 and sequencing of 348 colonies revealed the presence of three novel transcripts (Fig. 3c). Two of these were identical as detected by RNAseq; the third transcript found in only nine colonies harbors the c.351 donor that joins to a new splice acceptor at position c.539. All three transcripts resulted in sequence frameshift and occurrence of a premature stop codon at c.650. No new isoforms were found in P4–P7 by RNAseq; additional TA cloning and sequencing of cDNA in P5 and P6 detected only properly spliced full length transcripts.

### Synonymous variants are predicted not to affect RNA stability

The impact of synonymous variants on mRNA stability and secondary structure was determined using Mfold, RNAfold, and Quickfold tools. Synonymous substitutions were predicted not to significantly affect secondary structure of mRNA (Supplementary Fig. 4a). In addition, no relevant energy change (ΔG) was observed between Mut and WT (Supplementary Fig. 4b). As a comparison, five common synonymous polymorphisms from GnomAD and five nonsynonymous pathogenic *GATA2* mutations were included in the analysis. None of these variants had influence on the mRNA structure and thermodynamic characteristics.

### Analysis of protein stability and function

We investigated the levels of endogenous GATA2 protein in P9 who carried RNA-deleterious mutation c.351C>G, p.T117T and had sufficient primary specimen. Analysis was performed in patient-derived platelets since GATA2 was previously found to be highly expressed in this hematopoietic subpopulation (Supplementary Fig. 5) [33, 34].

**Fig. 4:**
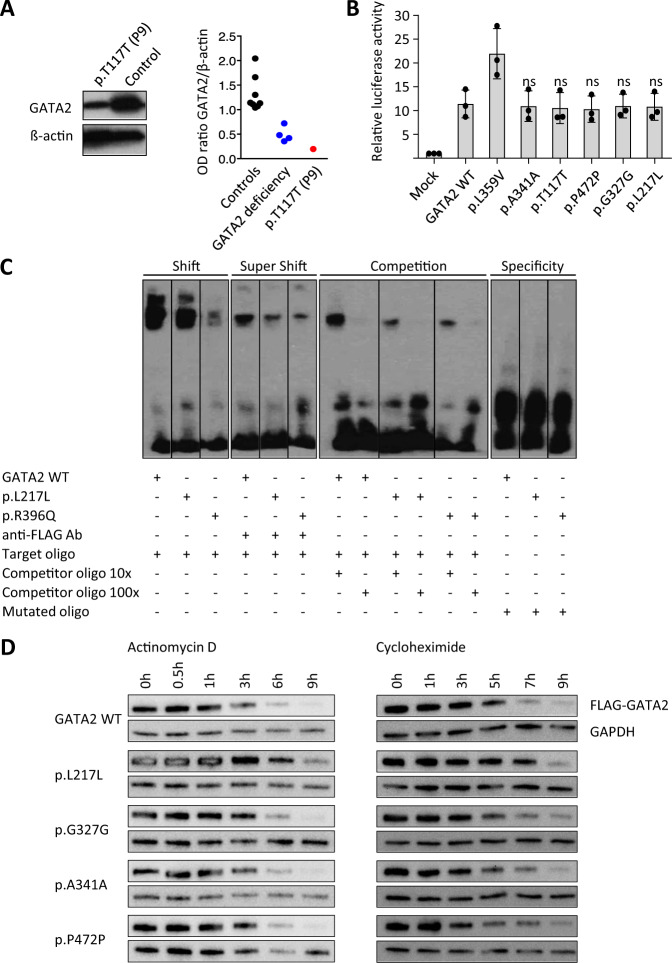
Assessment of protein expression and function. **a** Expression of endogenous GATA2 protein in platelets of P9 (c.351C>G, p.T117T). Left: Representative western blot image for the expression level of GATA2 and ß-actin. Right: Relative optical density of GATA2 protein normalized with ß-actin. GATA2 deficiency group comprises four pathogenic mutations in *GATA2* coding region. **b** Transactivation activity measured by GATA reporter assay. Previously reported p.L359V mutant was used as a positive control. Fold activation of luciferase reporter was determined from the triplicate values from three independent experiments and presented as the mean ± SD values. Comparison between the GATA2 WT and each synonymous Mut, as well as *p* value calculation was performed using a standard one-way ANOVA test. **c** The electrophoretic mobility shift assay (EMSA) depicting DNA-binding ability of GATA2 p.L217L, compared with WT and a known loss-of-function mutation p.R396Q, lanes spliced from the same gel runs (experiment repeated three times). Shift: semiquantitative comparison of GATA2 binding strength to the target oligo (biotin-labeled probe containing wild-type target sequence); Super Shift: specificity of FLAG-GATA2 binding to DNA sequence with anti-FLAG antibody (Ab); Competition: includes 10× and 100× excess of unlabeled wild-type probe competing for binding of the protein; Specificity: contains biotin-labeled probe with mutated target sequence which cannot be bound by the analyzed protein. Three latter setups verify that the signal obtained in the shift reaction is the result of specific DNA-protein interaction. **d** Assessment of translation efficiency (left) and protein stability (right) of GATA2 synonymous mutants in transiently transfected 293T cells treated with 1 µg/ml actinomycin D and 10 µg/ml cycloheximide, respectively. Experiments were performed in duplicates. Blots from representative experiments are shown.

GATA2 protein levels were severely reduced, similarly to other known pathogenic GATA2 mutations (Fig. 4a). Next, to determine the effect of the synonymous variants on GATA2 transcriptional function, GATA-specific reporter assay was performed. Transactivation activity was comparable between synonymous Mut and WT (Fig. 4b). We subsequently assessed the protein:DNA-binding ability using the electrophoretic mobility shift assay (EMSA) for the mutation c.649C>T, p.L217L. Using this limited approach, no significant difference in DNA binding between Mut and WT GATA2 proteins was seen (Fig. 4c). Of note, both functional experiments were performed at steady state with a high level of ectopic protein expression.

Because it is known that synonymous variants can impair translation, we aimed to analyze the effect of Muts on protein levels. We ectopically expressed cDNA under the principle that splicing effect will not be expected due to missing introns, and observed protein changes will result from altered translation. We blocked the transcription with actinomycin D in transfected 293T cells and analyzed protein levels over time (Fig. 4d). Expectedly, protein content decreased during the course of treatment for all genotypes resulting from exhaustion of mRNA reserves. However, p.L217L showed slightly higher protein content as compared with WT. To better delineate the cause for the relative increase in protein levels after transcription blockade, we then quantified the proteins after translation inhibition (cycloheximide). The p.L217L variant was associated with a slowdown of protein degradation visible after 5–7 h of treatment (Fig. 4d).

### Effect of synonymous *GATA2* c.649C>T variant on zebrafish hematopoiesis

**Fig. 5:**
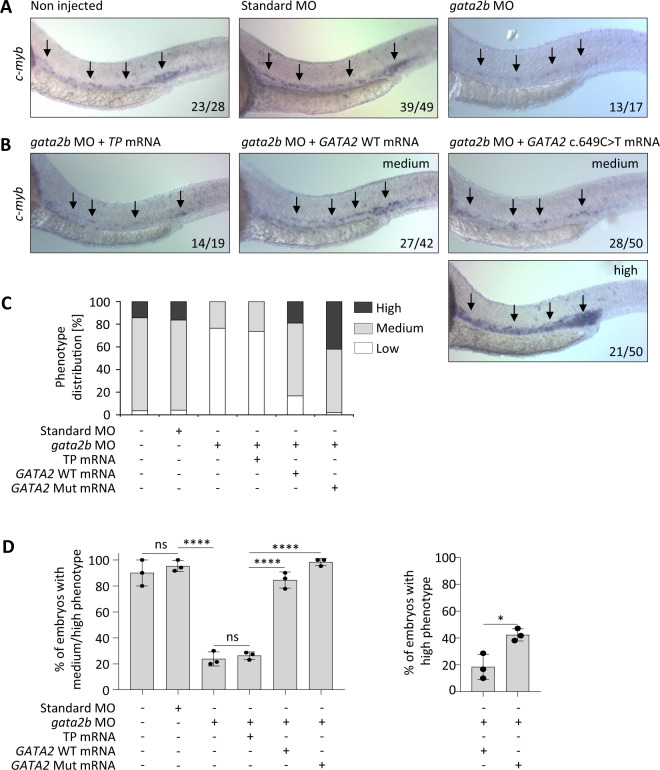
In vivo analysis of zebrafish hematopoiesis. **a** Whole-mount in situ hybridization (WISH) performed at 28 h post fertilization (hpf) for *c-myb* in embryos injected with control or *gata2b* morpholino (MO). The arrows indicate the location of hematopoietic stem and progenitor cells (HSPC) in the zebrafish aorta. The numbers in each picture indicate the number of embryos with depicted phenotype out of all embryos analyzed. **b** Rescue experiment performed on embryos co-injected with *gata2b* MO and human *GATA2* WT or Mut (c.649C>T) mRNA. Transposase (*TP*) mRNA was used as a random control RNA with no expected impact. A substantial number of the Mut (c.649C>T) mRNA-injected morphants show an even stronger (high) staining than control *TP* mRNA-injected siblings. **c** Graphical representation of the rescue experiment. Distribution of phenotypes in all tested animals: high, indicates a stronger staining to the one of noninjected and standard control MO-injected embryos; medium, indicates a staining equivalent or approximately equivalent to the staining of noninjected and standard control MO-injected embryos; low, indicates a weaker staining compared with the staining of noninjected and standard control MO-injected embryos (deficiency of HSPCs). **d** Left panel: Graphical representation of percentage of embryos with medium or high staining for each treatment category. Right panel: Percentage of morphant embryos depicting high staining when injected with either WT or Mut (c.649C>T) mRNA. Each experiment was performed in triplicates (*N* = 3; mean ± SD, Student’s *t* test, *p < 0.05, ****p < 0.0001).

For further analysis we selected the c.649C>T, p.L217L variant due to only partial reduction of the mutated allele expression in hematopoietic specimen of the P4. We hypothesized that the mutation may exert its effect on the protein level and aimed to determine if it alters zebrafish hematopoiesis. We used a previously published MO against *gata2b* [35] and visualized hematopoietic stem and progenitor cell (HSPC) in zebrafish embryos by whole-mount in situ hybridization of the HSPC marker *c-myb* at 28 h post fertilization, when HSPCs arise from the dorsal aorta. Expectedly, *gata2b* inhibition resulted in a reduction of HSPCs in zebrafish embryos (Fig. 5a, top right) [35]. We then performed a phenotype rescue experiment by co-injecting *gata2b* MO with human *GATA2* WT or Mut mRNA. Phenotype rescue (defined as medium/high phenotype; Supplementary Fig. 6) was achieved in 83% and 98% of embryos injected with WT and Mut mRNA, respectively, (Fig. 5b–d). However, we observed a significantly higher proportion of high phenotypes in animals rescued with Mut mRNA (42%) as compared with WT (19%), *p* < 0.05 (Fig. 5d right panel).

## Discussion

GATA2 deficiency is a monogenic disorder known so far to be caused by heterozygous nonsynonymous mutations, whole gene deletions or intronic enhancer mutations, all of which result in haploinsufficiency. In this study, we report the identification of synonymous, RNA-deleterious mutations in *GATA2* that accounted for 8.2% of all *GATA2*–mutated patients and 14.8% of cases with *GATA2* exonic substitutions. In total, we identified nine patients harboring five distinct synonymous *GATA2* variants that are either absent or exceedingly rare in general population: p.T117T, p.P472P, p.L217L, p.G327G, and p.A341A. Two of these (p.T117T and p.P472P) were encountered in multiple unrelated pedigrees, suggesting either independent mutational events or rare founders in the European population (which is possible at least for p.P472P present in gnomAD in 23 individuals of non-Finnish European ancestry). The phenotype of patients carrying synonymous variants resembled GATA2 deficiency. All of the patients were alive at last follow-up with exception of one patient who died from HSCT-related complications. Additional mutations in *GATA2* were not identified. Other MDS-predisposing conditions were excluded based on clinical studies and WES/WGS in all patients with the exception of P6 who carried two VUS in the *SAMD9* gene. No specific features demarcating GATA2 from SAMD9 syndrome were present in this patient; hypospadias are unspecific and had been reported in both conditions [36, 37]. At this point, we cannot rule out that in P6 both gene defects acted in a synergistic manner facilitating MDS development.

Computational prediction assigned an increased probability of missplicing to three of the five variants. Further assessment of mutation deleteriousness with existing *in silico* tools failed to ascribe pathogenic effects. Because of the difficulty in predicting deleteriousness, synonymous mutations have been generally left out in genomic studies. However, it is likely that many disease-causing mutations are being consistently overlooked—including mutations located in noncoding regions of the genome as well as synonymous variants. So far, little is known about the role of such mutations in hematopoietic malignancies due to lack of routine screening of the inter-/intragenic regions. Besides known recurrent deleterious mutations in the regulatory element of *GATA2* [10] there are only few examples of noncoding mutations associated with BMF. Recently, two patients were reported with dyserythropoietic anemia and an intronic substitution in *GATA1* gene that is 24 nucleotides upstream of the canonical splice acceptor site. This alteration resulted in reduced canonical splicing and increased use of an alternative splice acceptor site that causes a partial intron retention event [38]. Moreover, mutations in 5’UTR and deep intronic region of *ELANE* gene have been reported to be associated with severe congenital neutropenia [39]. Due to lack of studies integrating functional evaluation, the prevalence of such variants in Mendelian disorders is yet to be determined. It is remarkable that recent pancancer studies report acquired synonymous driver mutations at a rate of ~6–8% among all single-nucleotide changes found in human cancers [40]. This is strikingly similar to the proportion of (germline) synonymous mutations identified in our study. Mutations causing phenotypically severe hereditary disease are mainly introduced as random de novo events, and it is well accepted that purifying selection will eventually eliminate these deleterious alleles. This is especially valid in high-penetrance conditions, such as GATA2 deficiency that often manifests before the reproductive age and thus results in reduced fecundity.

There are multiple ways how synonymous substitutions can exert deleteriousness even though the amino acid sequence is not changed. As confirmed using three orthogonal approaches, all of the mutations found here resulted in a nearly complete and selective loss of the Mut transcript in hematopoietic cells, with the exception of c.649C>T, p.L217L that showed Mut allele reduction to ~20%. In contrast, paired analysis of two patients revealed a higher Mut allele expression in skin fibroblasts versus hematopoietic cells. Potential explanations for this discrepancy might be the variability of allelic expression across different tissues [41, 42] or the notion of context-dependent monoallelic expression observed for ~20% of human genes [43]. In addition, we observed that divergence in allelic ratio depends not only on the tissue analyzed but also on the stage of RNA processing. Strikingly, the mutation frequency in BM of two patients (c.351C>G and c.1023C>T) increased from nearly absent in mRNA to 30% in total RNA transcripts, implying that the defect manifests at a late stage of RNA maturation, at least for these two variants. Splicing disruption was predicted for three variants (c.351C>G, c.981G>A, and c.1023C>T); however, splicing analysis confirmed novel splicing pattern only for c.351C>G. This mutation resulted in aberrant transcripts with premature stop codon which makes it functionally equivalent to a frameshift-truncating mutation causing nonsense-mediated decay. For the remaining four mutations, no abnormal splicing was detected. It is conceivable that these Mut mRNAs are extremely unstable and subjected to a very rapid sequestration. Another potential explanation for loss of allelic expression is epigenetic silencing that could arise from aberrant promoter methylation. Supporting this, allelic disbalance due to hypermethylation was recently observed in one patient with GATA2 p.T354M mutation [44]. Synonymous variants can also affect translation and thus result in increased or decreased protein stability or function. Surprisingly, p.L217L Mut protein was slightly more stable in vitro, although its function (tested in vitro using the EMSA DNA gel shift assay at steady state, with ectopic *GATA2* overexpression) seemed not to be affected. Further, this mutation not only rescued the GATA2-deficient phenotype in zebrafish, but also resulted in a significantly higher number of HSPCs in comparison with control animals. Higher stability of this mutated protein might potentially explain the relative increase in its functional properties in vivo. In analogy, it is known that moderate *GATA2* overexpression enhances proliferation and self-renewal of progenitor cells [45]. We reason that the more efficient rescue of the morphant phenotype can be associated with higher stability of the p.L217L Mut, which is seen when transiently overexpressed in 293T cell line (Fig. 4d). Because of the challenging data (decrease of Mut RNA expression but higher protein stability of protein) we do question the pathogenicity of this mutation until additional biological data or patients are reported. Limited availability of patients’ primary specimens as well as instability of the transcripts with synonymous mutations precluded further mechanistic studies.

Reported diagnostic yields for WES/WGS in single individuals can reach ~40% and heavily rely on computational predictions [46, 47] which are difficult to achieve for synonymous mutations. Moreover, WES is limited to the analysis of coding regions only. Even though genome sequencing overcomes this constraint, it generates an enormous output of alterations within coding and noncoding regions of the genes. In setting of GATA2 deficiency, WGS would facilitate the detection of pathogenic intronic mutations in regulatory region in intron 4 (corresponding to +9.5 kb enhancer region) as well as whole gene and partial gene deletions. However, allelic loss on RNA level would be missed. The utility of transcriptome analysis was previously highlighted by the identification of disease-causing mutations in patients with negative exome or genome sequencing results, increasing the diagnostic rate by as much as 35% [48, 49]. Hence, we propose that diagnostic sequencing should incorporate a cascade approach where RNA sequencing follows inconclusive DNA analysis in patients with suspected disease. This approach is feasible not only for patients with GATA2 deficiency but also in patients with high index of suspicion for a specific Mendelian disorder but without a known pathogenic mutation. Our findings suggest that a straightforward Sanger or deep sequencing of cDNA would be sufficient to confirm the RNA-deleteriousness of a synonymous variant.

In summary, we demonstrate that a significant proportion of GATA2-deficient patients carry damaging synonymous alterations. These genetic changes, previously excluded from analysis due to their likely silent effect, should be incorporated into standard diagnostic pipeline for individuals with GATA2 disease phenotype. However, patients with other hereditary BM failure and MDS syndromes might also benefit from this extended diagnostic approach. In the long term, identification of pathogenic synonymous variants has the potential to improve genetic counseling, HSCT donor selection, and clinical outcomes.

## Supplementary information

Supplementary Material

## Data Availability

WES data have been deposited at the European Genome–phenome Archive (EGA), which is hosted by the EBI and the CRG, under accession number EGAS00001003817. Further information about EGA can be found on https://ega-archive.org “The European Genome–phenome Archive of human data consented for biomedical research”.
