# Supplementary material for: Synonymous GATA2 mutations result in selective loss of mutated RNA and are common in patients with GATA2 deficiency
Source: Leukemia. 2020 Jun 18;34(10):2673–87. doi: 10.1038/s41375-020-0899-5 (PMC7515837; doi:10.1038/s41375-020-0899-5)
Supplement: Supplementary file 1 — Supplementary Material [file 41375_2020_899_MOESM1_ESM.docx]

**SUPPLEMENTAL INFORMATION**

Page:

Phenotype of patients with synonymous *GATA2* mutations 1

Supplemental Methods 4

Supplemental Figure 1 8

Supplemental Figure 2 9

Supplemental Figure 3 10

Supplemental Figure 4 11

Supplemental Figure 5 12

Supplemental Figure 6 13

Supplemental Table 1 14

Supplemental Table 2 15

References 16

**PHENOTYPE OF PATIENTS WITH SYNONYMOUS *GATA2* MUTATIONS**

**Patient P1** (D 1239) presented at the age of 12 years with inflammatory disease characterized by fever, chronic cough, edema, bursitis, tenosynovitis, arthritis, lymphadenopathy and pericardial effusion; labs showed hypergammaglobulinemia, increased blood sedimentation rate and CRP; antinuclear antibodies were negative. The patient showed severe normocytic hypochromic anemia necessitating blood transfusion. The direct antiglobulin test was positive and warm antibodies were identified. Due to positive family history in the mother who presented with GATA2-deficient phenotype (immunodeficiency, severe infections and lung disease (1)), bone marrow analysis was performed and revealed normocellular refractory cytopenia of childhood (RCC) with monosomy 7. In line with this diagnosis, B-cell lymphopenia, monocytopenia and lack of plasmacytoid dendritic cells were present. The further disease course was complicated by chronic arthritis treated with steroids, pneumocystis pneumonia and mild thrombocytopenia. The patient underwent allogeneic hematopoietic stem cell transplantation (HSCT) from an HLA-matched unrelated donor (conditioning with treosulfan, thiotepa, fludarabine). At the last follow up, the patient was 13.5 years old and alive with 100% donor chimerism.

**Patient P2** is the younger sister of P1. As a potential sibling donor, she was tested positive for the same *GATA2* mutation. At the last follow up at age of 12 years, she had an uneventful history without cytopenia or inflammatory symptoms. Laboratory analysis revealed mild monocytosis (11.7%) and mild hypogammaglobulinemia for IgG and IgA with normal IgM. Specific vaccination-induced antibodies were positive for measles but negative for tetanus. Lymphocyte immunophenotyping revealed NK and B-cell lymphopenia with reduced numbers of naïve and transitional B cells.

**Patient P3** (D 749) showed first signs of disease at the age of 14 years, when she reported recurrent headache, hissing noises in the ear and fatigue. She had pancytopenia with macrocytosis (MCV 105 fL), and based on bone marrow exam was diagnosed with myelodysplastic syndrome with excess of blast (MDS-EB) and monosomy 7 in 12 of 20 metaphases. The patient was dependent on erythrocyte and platelet transfusions and suffered from recurrent aphthous ulcerations. After six months, the disease progressed to MDS-related acute myeloid leukemia (AML) with 75% blast cells in bone marrow. Initial treatment with high-dose cytarabine and mitoxantrone was followed by HSCT. Due to the lack of an HLA-matched donor, cord blood CD34+ cells were used as graft. The disease course was complicated by pulmonary aspergillosis; skin GvHD and drug intolerances (i.e. vancomycin, metoclopramide, voriconazol). Four months after HSCT, the patient suffered from systemic adenoviral infection with hepatic failure, pericardial effusion, hypertensive crisis with epileptic seizure and bleeding. Macrophage-activation syndrome was suspected. The patient died due to multiorgan failure at the age of 15.4 years.

**Patient P4** (LT) was first diagnosed with thrombocytopenia (62,000/µL) and monocytosis (39.6%) at the age of 3 years. Retrospective review showed that monocyte counts were elevated since birth (range 20-40%). Bone marrow analysis showed dysplastic megakaryopoiesis, mild dysplasia of erythropoiesis and granulopoiesis and numerous immature B cells but no increase in blast counts. After exclusion of Fanconi anemia, RCC was established as diagnosis. Cytogenetic analysis on bone marrow cells revealed monosomy 7. Non-hematological problems included hypoxia during birth requiring C-section as well as transient acute renal insufficiency and respiratory insufficiency in the neonatal period. At presentation, he showed low set ears, flat nose, several areas of skin hypopigmentation and joint hypermobility. The *GATA2* mutation was excluded in the patient’s brother who served as donor for HLA-matched HSCT. The patient was alive and well at the last follow up, 2.4 years after HSCT.

**Patient P5** (D 722) suffered from his 7^th^ year of life from macrocytic anemia with recurrent hemolytic episodes with jaundice. Occasionally, he also showed leukopenia (WBC >1,000/µL) and mild thrombocytopenia (platelets >100,000/µL) but bone marrow exams remained inconclusive. Vitamin B12 and folate deficiencies as well as spherocytosis and PNH disease were excluded but direct antiglobulin test was intermittently positive, leading to the diagnosis of autoimmune hemolytic anemia (AIHA). At the age of 11 years, he showed hepatosplenomegaly (4 cm below costal arch) and reported fatigue. Bone marrow analysis revealed dysplasia of all lineages with hyperplastic erythropoiesis, and the patient was diagnosed with RCC. No cytogenetic aberrations were found in bone marrow cells. Until the last follow up at the age 18.5 years he showed stable disease without the need of transfusions.

**Patient P6** (D 1142) presented with macrocytic anemia, thrombocytopenia, leukopenia and severe neutropenia (ANC 385/µL) at the age of 11.5 years. Bone marrow examination revealed hypocellular RCC with monosomy 7. Non-hematological problems included hypospadias. Allogenic HSCT from HLA-matched unrelated donor was performed at the age of 12 years. Patient exhibited good regeneration of hematopoietic system 18 months after transplantation and was alive and well at the last follow up at the age 15.2 years.

**Patient P7** (D) presented at the age of 14 years with thrombocytopenia and microcytic anemia. In addition, she was suffering from selective mutism and hypermenorrhea. Thrombocytopenia was interpreted as idiopathic thrombocytopenia (ITP) and showed good response to treatment with steroids and immunoglobulins (platelet increased from 10,000/µL to 376,000/µL). Due to iron deficiency the patient received oral iron supplementation. Bone marrow aspirate was suspicious for RCC with mild dysplasia in all three blood cell lineages. No cytogenetic abnormality was found, and no follow up marrow examinations were performed. At the last follow at the age of 20.9 years the patient was well and did not require supportive care.

**Patient P8** (I 386) presented at the age of 4 years with thrombocytopenia, not responding to both immunoglobulin infusion and steroids given for suspected ITP. Bone marrow aspirate showed a marked reduction in the number of megakaryocytes; both the erythroid and myeloid lineages were normal. In the following weeks, she had progressive neutropenia and developed a hepatic abscess. Moreover, this patient became transfusion-dependent for erythrocytes. The findings of bone marrow exams were consistent with a diagnosis of RCC with normal karyotype and the chromosomal breakage test was negative. The patient received matched unrelated donor HSCT. Sustained donor cell engraftment was achieved; the girl developed grade II skin-only acute GvHD that was successfully treated with steroids and extra-corporeal photochemotherapy. Twelve months after transplantation, the patient is alive and disease-free without any sign of chronic GvHD with 100% donor chimerism.

**Patient P9** (UKA2604) presented at the age of 24 with bronchiectasis complicated by mycobacterium avium complex, Crohn’s colitis, HPV-driven anal, vulval and cervical intra-epithelial neoplasia (stage III). She had mild pancytopenia with absolute deficiency of dendritic cells, monocytopenia, B and NK-cell lymphopenia and elevated Flt3 ligand of 11,400 pg/ml. Bone marrow aspirate and biopsy showed evidence of MDS with multilineage dysplasia, but no cytogenetic abnormality was detected. The patient received a 9/10 HLA-matched unrelated donor transplant aged 27 years. She is currently alive and GVHD-free with 100% myeloid and T cell engraftment 5 years later. HPV-driven neoplasia has resolved or regressed to stage I and not required further therapy. The transplantation procedure of this patient was previously described (2).

**SUPPLEMENTAL METHODS**

**Targeted sequencing of genomic DNA and cDNA**

Sequencing of 5 coding exons and intron 4 containing conserved EBOX-GATA-ETS regulatory region (+9.5kb) of the *GATA2* gene (NM_032638.4) was accomplished using targeted deep sequencing (AmpliSeq custom panel IAD51150_130, Thermo Fisher Scientific) and Sanger sequencing for validation. Germline origin of identified variants was verified in DNA extracted from skin fibroblasts and/or purified lymphocytes as previously reported (3). Deep sequencing was performed on the MiSeq Sequencer (Illumina, USA) after PCR-based enrichment and library preparation using the NEBNext® Ultra™ II DNA Library Prep Kit for Illumina (NEB). The obtained text-based files (FASTQ) were processed with SeqPilot/SeqNextq (v.4.40) for quality assessment and variant annotation. A quality score of 30 (Q30, 99.9% base call accuracy) and a minimum absolute coverage of 50 reads was implemented to abate incorrect base calls. A minimum coverage of 150X was employed for variants with a minimum variant allelic frequency (VAF) of 5%. The quality thresholds were recommended by the Illumina Technical Notes: 1) Quality Scores for Next-Generation Sequencing, 2011, 2) Somatic Variant Caller, 2012. Variants were visualized using SeqPilot/SeqNextq (v.4.40) in addition to Alamut Visual (v.2.8-2.10, Interactive Biosoftware, Rouen, France).

The classification of variants was subjected to our in-house hierarchical approach. Population frequency (gnomAD, ESP, 1000g): Pathogenic range; novel (absent in population), very rare (minor allele frequency, MAF<0.01%), rare (0.01%<MAF<0.1%). SNP; frequent (MAF≥0.1%) and very frequent (MAF≥0.5%). Quality Assessment (QA): Variants VAF<5%, depth<30x and <2x in each forward and reverse in alternative allele where not considered. Mutation Type: Pathogenic range; deletion (in-frame, whole gene), splicing, stop gained (frameshift, nonsense). Undefined; missense and synonymous. Computational predictors: Meta-scores; CADD, DANN, REVEL. SNV-evaluation; SIFT, Polyphen-2, LRT, FATHMM, PROVEAN, VEST3, Mutation taster, Mutation Assessor, MetaLR, MetaSVM. Conservation; Genomic Evolutionary Rate Profiling (GERP++RS) was calculated using ANNOVAR (4), Phylop, PhastCons, SiPhy. Clinical observations (clinical presentation, karyotype, MDS type): pathogenic range; previously reported, familial, known phenotype. Undefined; unspecific phenotype. Patient 8 was sequenced using targeted bone marrow failure and MDS panel (custom Roche SeqCAp EZ Hyper Cap Library capture kit, Roche, Switzerland) on a MiSeq platform (Illumina, USA). Filtering and prioritization of variants from this panel was performed using an in-house implemented pipeline as previously reported (5, 6).

**Whole exome sequencing (WES)**

WES was performed for patients P1, P3, P5, P6 and P7 in bone marrow granulocytes (BM-GR) as well as skin fibroblasts of P4. WES procedure and variant filtering were previously described (7). Briefly, exon region capture was accomplished with Agilent SureSelect v5.0 (Agilent, Santa Clara, CA) and library was prepared using TruSeq (Illumina). Paired-end (2x100bp) sequencing was performed on HiSeq2000 instrument (Illumina). The analysis focused on 300 genes associated with predisposition to bone marrow failure and hematopoietic malignancies (Fanconi anemia, Dyskeratosis congenita, congenital neutropenia, DBA, hereditary MDS and myeloid neoplasia, congenital thrombocytopenia, chromosomal instability and mismatch repair-deficiency, RASopathies), and those reported in Pan-Cancer studies with hematopoietic cancers (8-13).

**Targeted investigations of *GATA2* transcript expression**

Total RNA was extracted from peripheral blood, bone marrow or fibroblasts using the TRIzol reagent (Thermo Fisher Scientific). RNA from patients (P1, P3 – P7) and 3 healthy controls was subjected to reverse transcription (QuantiTect Reverse Transcription kit, Qiagen) using oligo(dT)_18nt_ priming (for P1, P3, P6 and P7) or a mix of oligo(dT)_18nt_ and random hexamers (P4, P5). In order to compare the efficiency of different priming methods, cDNA of P1 and P6 was additionally synthesized with random hexamers only. P9 cDNA RT-PCR was performed as follows: cDNA from patient-derived fibroblasts was reverse transcribed with random hexamers (RevertAid First Strand cDNA Synthesis Kit, Thermo Fisher Scientific) and then amplified using primers spanning exon 2 – exon 5 of the *GATA2* gene (supplemental Figure 1 and supplemental Table 2).

Initially, in order to evaluate the expression of wild type (WT)/ mutated (Mut) *GATA2* alleles, Sanger sequencing of cDNA was performed in 7 of 9 cases with synonymous alterations and 3 healthy controls. Results were confirmed in at least 2 independent experiments. Deep sequencing was used to precisely quantify the loss of mutant mRNA in 6 patients (supplemental Table 2). The presence of novel transcripts was assessed using direct sequencing of expected and alternative PCR products resolved on agarose gel. To analyze single alleles independently TA cloning and sequencing was performed as previously described (14). Briefly, PCR products amplified from patient-derived cDNA using *GATA2* specific primers were gel purified (Wizard SV Gel and PCR Clean-Up kit, Promega, Mannheim, Germany), ligated into TA cloning vector pCR2.1 (Thermo Fisher Scientific) overnight at 16°C and transformed into TOP10 E.coli. Multiple colonies were amplified by colony PCR using M13 primers. PCR products were purified, and Sanger sequenced.

Analysis of *GATA2* expression in different hematopoietic cells of healthy controls was performed as follows: RNA was isolated using the AllPrep® DNA/RNA/Protein Mini kit (Qiagen), and cDNA was generated with RevertAid H Minus First Strand cDNA synthesis kit (Thermo Scientific). *GATA2* expression was quantified by qPCR with TaqMan gene expression assay for *GATA2* (Hs00231119_m1; Life Technologies) and *GAPDH* (4352934E; Life Technologies) according to manufacturers’ instructions using an ABI 7900HT Fast Real-Time PCR System. Relative quantification of the mRNA levels was performed using the ΔCT method with *GAPDH* as the reference gene.

**Structural modeling of *GATA2* mRNA**

*In silico* modeling was performed to assess the effect of synonymous variants on RNA stability and conformation. Control groups consisted of 5 synonymous variants with high allelic frequency in the gnomAD population: c.15C>G, p.P5P: 66.24% (154544: alternative alleles /233310: total alleles); c.564G>A, p.T188T: 4.17% (11524/276670); c.1233G>A, p.A411A: 2.17% (6012/277196); c.66C>G, p.P22P: 0.08% (205/267714); c.114G>A, p.Q38Q: 0.08% (207/271388) and 5 pathogenic non-synonymous *GATA2* mutations: c.1061C>T, p.T354M; c.1075T>G, p.L359V; c.1081C>T, p.R361C; c.1186C>T, p.R396W; c.1187G>A, p.R396Q. Initially, the *GATA2* WT mRNA and all associated Mut nucleotide sequences (FASTA) were uploaded to Mfold, RNAfold and Quikfold servers (15-17). They predict the most energetically stable secondary structure of mRNA based on calculated minimum free energy (ΔG) values. ΔG is defined as the sum of the free energies assigned to all the loops and stem regions. The lower the energy value, the higher the stability of predicted secondary structures.

**GATA2 splicing analysis using RNA sequencing**

RNA was extracted (Quick-RNA MicroPrep kit, Zymo Research, Freiburg, Germany) from flow-sorted CD34+ cells (CD34-APC antibody #345804 BD Biosciences, Heidelberg, Germany) from BM of P1, P4-P7. Libraries enriched with polyadenylated transcripts were prepared using SMART-Seq® v4 Ultra® Low Input RNA Kit (Takara, Saint-Germain-en-Laye, France) and Nextera XT DNA Library Prep Kit (Illumina) and paired-end (2x150bp) sequenced on a NovaSeq 6000 (Illumina). Identification of novel *GATA2* transcripts and Sashimi plot generation was performed using HISAT/StringTie and Ballgown suite (18). First, the reads were mapped to the reference genome, assembled, quantified and merged across all samples using StringTie. Next, transcript abundances were re-evaluated in order to create an input file to Ballgown for expression analysis.

**Sequence analysis**

Unless otherwise indicated, all sequences were uniformly analyzed using SeqNext and SeqPilot (JSI Medical Systems). Nucleotide conservation alignment was performed with Nucleotide BLAST (https://blast.ncbi.nlm.nih.gov/). Putative *de novo* splicing effect of the 5 synonymous alterations was assessed by splicing tools integrated in Alamut Visual v2.10.0 (Interactive Biosoftware) and Human Splicing Finder v3.0 (19) which combines 12 different algorithms to predict the effect of mutations on identified splicing motifs. The gnomAD population cohort (20) was used to assess allele frequencies of selected variants in a population of >120 000 individuals.

**GATA2 expression studies**

The pFLAG-CMV2-GATA2 construct was purchased from Addgene (#1418). Synonymous *GATA2* mutations were introduced using QuickChange Lightning Site-Directed Mutagenesis Kit (Agilent Technologies). FLAG-tagged GATA2 protein was ectopically expressed from the aforementioned construct in 293T cell line. Cells were cultured in Dulbecco’s Modified Eagle’s Medium (DMEM) supplemented with 10% Fetal Bovine Serum (FBS), 2 mM L-glutamine and 1% Penicillin/Streptomycin. 293T cell line was purchased from Sigma-Aldrich and are negative for mycoplasma as assessed by conventional PCR using Venor GeM Classic kit (Minerva Biolabs, Berlin, Germany). Protein content was assessed after treatment of the transiently transfected cells with 1 µg/ml actinomycin D and 10 µg/ml cycloheximide, followed by Western blotting with anti-FLAG antibody (mouse monoclonal, clone M2, F1804; Sigma-Aldrich, Hamburg, Germany). Detection of endogenous GATA2 protein in patient-derived platelets was accomplished using anti-GATA2 antibody (mouse monoclonal, clone 2D11, H00002624-M01; Novus Biologicals, Oxfordshire, UK).

**Luciferase reporter assay**

Transactivation activity of GATA2 protein was measured using reporter assay in 293T cells co-transfected with a pFLAG-CMV2-GATA2 (GATA2 WT, p.A341A, p.T117T, p.P472P, p.G327G, p.L217L and p.L359V as a gain-of-function control), GATA luciferase reporter vector (Affymetrix, Milano, Italy), and pRL (Renilla luciferase) using Attractene Transfection Reagent (Qiagen). Luciferase activity was measured in cell lysates (TECAN reader) 24 h post-transfection using a dual luciferase assay system (Promega). Luminescence values were normalized based on constitutively expressed Renilla luciferase. Relative luciferase activity was determined from triplicate measurements obtained from 3 independent experiments.

**Electrophoretic mobility shift assay**

DNA-binding ability of GATA2 Mut protein p.L217L was investigated in 293T cell line by EMSA. Nuclear lysates were prepared 24 h post-transfection, then incubated with the oligonucleotide containing GATA recognition sites and the assay was performed according to manufacturer‘s instructions (Active Motif). The previously reported pathogenic p.R396Q mutation was used as an assay control.

**Zebrafish studies**

Zebrafish (Danio rerio) strain Tübingen was maintained in the animal facility of the Max Planck Institute of Immunobiology and Epigenetics. The sample size was chosen as previously described (21). No animals were excluded, and no randomization was applied. Only 1–5 days post fertilization (dpf) embryos were used in this study and sex was not determined at these stages. Embryos were injected (PV820 Pneumatic PicoPump, World Precision Instruments, Friedberg, Germany) at one-cell stage with 24 ng of standard morpholino (MO) or *gata2b* splicing anti-sense MO as previously described (22). Stock solutions were diluted as recommended by the manufacturer. The sequence for the *gata2b* splicing anti-sense MO was: 5'-TTCACGTCCTATTGGCACACGATGC-3', and standard oligo: 5′-CCTCTTACCTCAGTTACAATTTATA-3′. For the rescue experiments, one-cell stage embryos were injected with MO and 50 pg of WT or Mut *GATA2* mRNA, (synthesized by *in vitro* transcription from pcDNA3-GATA2 DNA constructs using mMessage mMachine High Yield Capped RNA transcription T7 kit according to the manufacturer’s instructions, Ambion, Oberursel, Germany). Equal expression of GATA2 WT and Mut from each plasmid construct used for mRNA generation was confirmed by western blot on 293T cells transiently transfected with pcDNA3-GATA2 (supplemental Figure 6B). Injected embryos were grown until 28 hpf, dechorionated with pronase (Sigma-Aldrich) and fixed in 4% paraformaldehyde (PFA). Whole-mount in situ hybridization (WISH) was performed as previously described (23) with minor modifications.

**Whole-mount *in situ* hybridization (WISH)**

The *c-myb* probe was used (24). Digoxigenin-labeled RNA probe was synthesized by *in vitro* transcription with T7 RNA polymerase (Thermo Fisher Scientific). The embryos were treated with 1-Phenyl-2-Thiourea (PTU) solution to prevent melanin synthesis. Subsequently, they were dechorionated and fixed in 4% PFA overnight (4^o^C). After fixation, the embryos were washed and dehydrated in 100% methanol at 4^o^C overnight. Next, they were rehydrated, permeabilized with proteinase K and re-fixed in 4% PFA for 20 min at room temperature (RT). Washing in PBS-T and incubation in hybridization buffer (50% formamide, 5xSSC, 0.1% Tween20, ddH2O) at 70°C for 30 min was performed. Hybridization solution containing 1 ng/µL of the *c-myb* RNA probe was then added and the embryos were incubated at 70°C overnight. After serial washes, blocking solution (PBS-T, 2% sheep serum, 2 mg/mL BSA) was added for 30 min at RT. Then, the embryos were treated overnight at 4^o^C in blocking solution containing 0.15 U/µL anti-digoxigenin antibody (Roche). Finally, they were washed and incubated in staining solution at RT (for 10 mL of staining buffer: 35 µL 50 mg/mL BCIP, 45 µl 50 mg/mL NBT) until stained. All WISH experiments were performed on at least three separate occasions. Embryos were imaged in 100% glycerol, using a Leica stereomicroscope. Qualitative scoring (number of embryos with altered HSCs per number scored) of WISH staining was conducted manually by visual observation, blindly, independently by two people. The numbers on each picture in Figure 5A and B represent the number of embryos that exhibit presented phenotype relative to the total number of imaged embryos. Low, medium and high phenotype of morphants was distinguished based on the amount of HSPCs in comparison to the non-injected and standard control MO-injected embryos: High: stronger staining to the one of non-Injected and standard control MO-injected embryos; indicates enhanced number of HSPCs; Medium: staining equivalent or approximately equivalent to the staining of non-Injected and standard control MO-injected embryos; indicates normal/ rescued phenotype; Low: weaker staining compared to the staining of non-injected and standard control MO-injected embryos; indicates deficiency of HSPCs.

**SUPPLEMENTAL FIGURES AND TABLES**


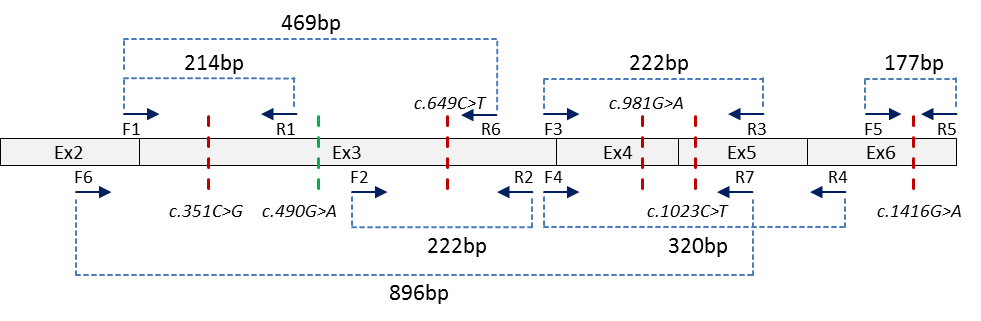


**Supplemental Figure 1 (linked to Figure 2 and Supplemental Table 2). Strategy of cDNA PCR amplification for Sanger and deep sequencing (if applied)**. Red dashed lines: location of synonymous *GATA2* mutations; green dashed lines: location of a common *GATA2* non-synonymous polymorphisms; arrows indicate the position of the primers (not scaled).

**
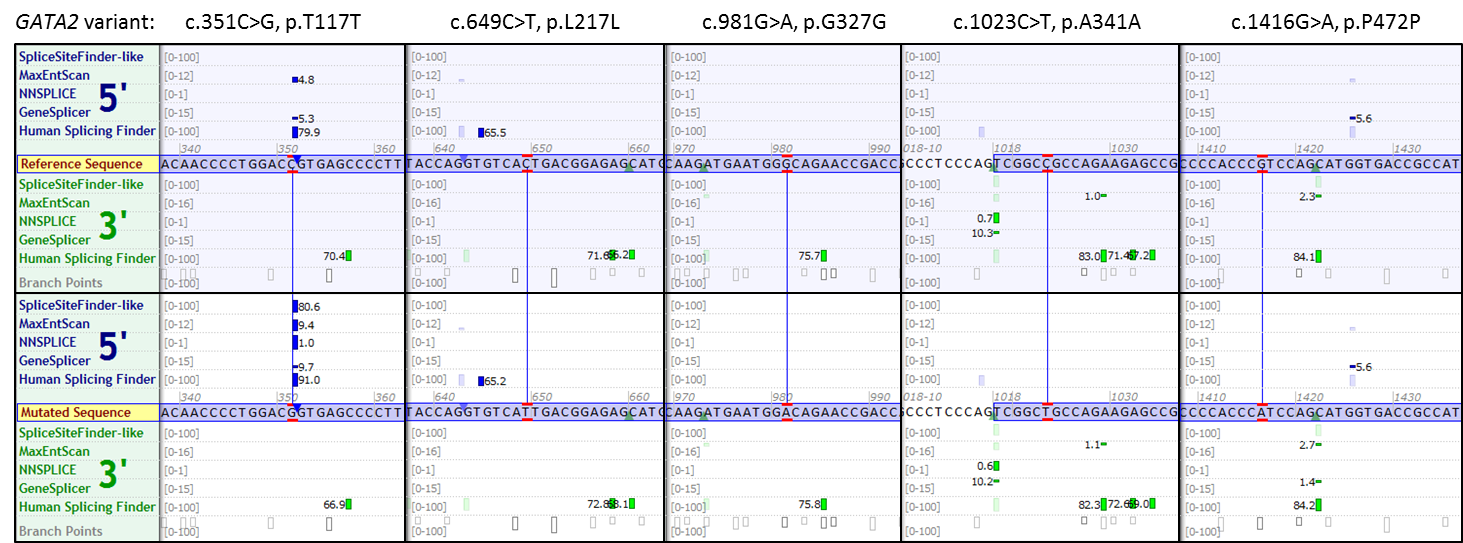
**

**Supplemental Figure 2 (linked to Figure 1). Splicing prediction for the *GATA2* synonymous mutations from Alamut Visual software.** Multiple algorithms listed on the left side of the figure score the probability of each nucleotide to act as a splicing donor (5’) or acceptor (3’). Comparison between reference (top) and mutated (bottom) sequence is shown. As depicted, C>G substitution increases the chance of a splicing donor activation in a position c.351. No significant changes are caused by other variants.

**
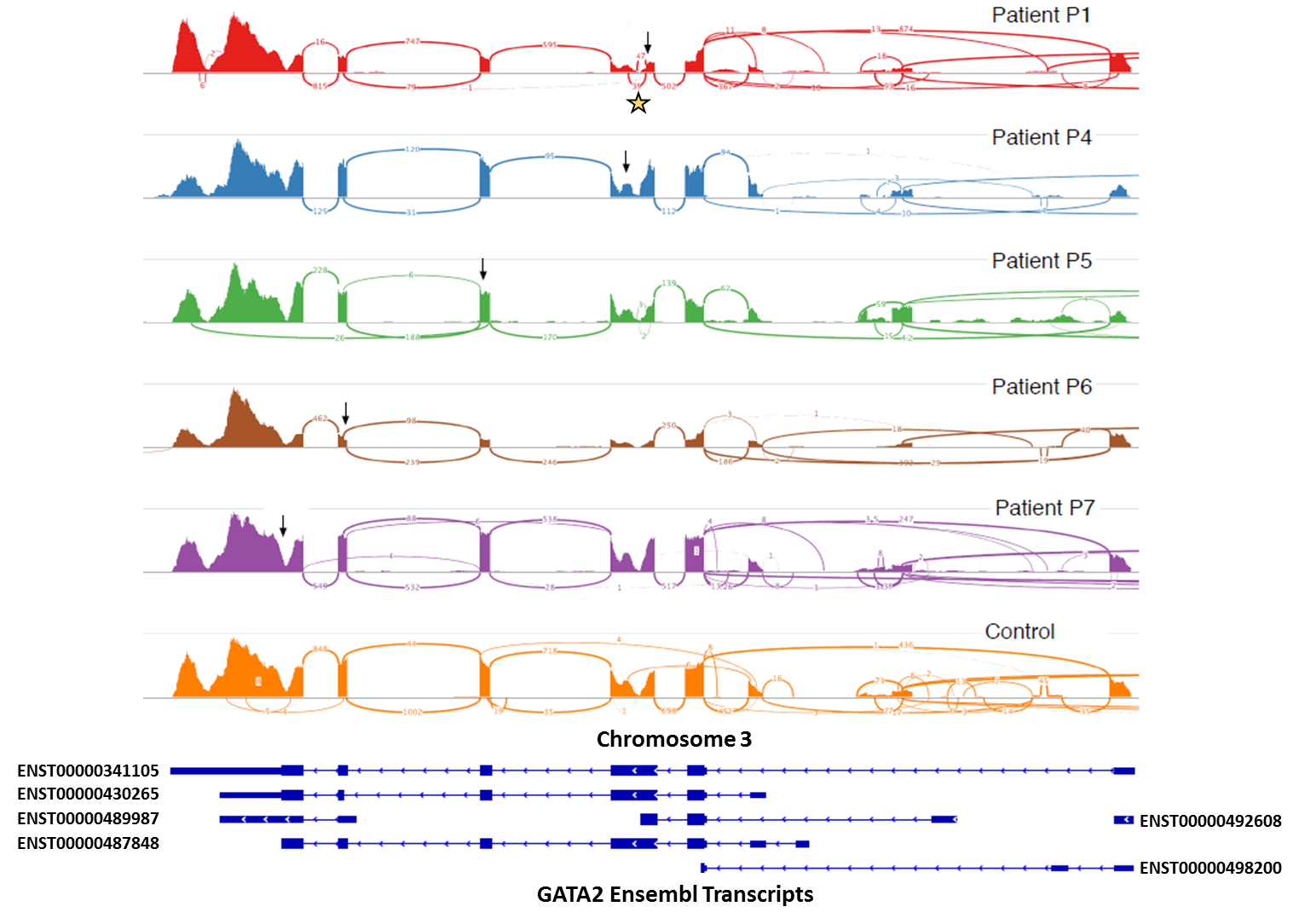
**

**Supplemental Figure 3 (linked to Figure 3). Splicing analysis of transcripts obtained from RNA sequencing.** Sashimi plots visualizing splice junctions along genomic coordinates of *GATA2* gene (located on the reverse strand with ENST00000341105 as a canonical transcript, Refseq: NM_032638.4). Black arrows specify the position of synonymous mutations. Yellow star indicates the alternative splicing junctions in P1 (39 and 47 reads).


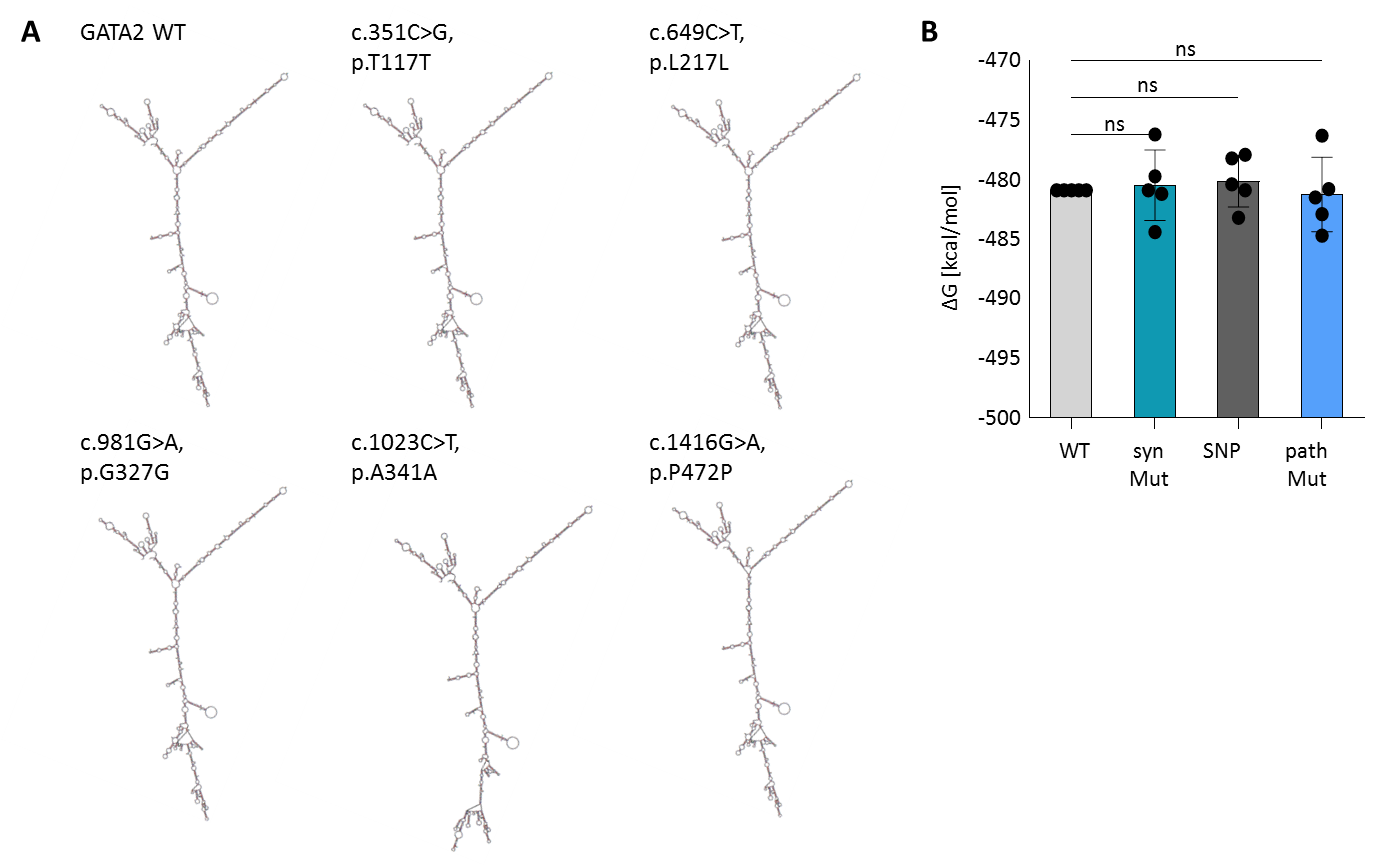


**Supplemental Figure 4. Secondary structure and thermodynamic characteristics of *GATA2* mRNA.** (A) Structure of GATA2 wild WT and Mut mRNA predicted using Mfold tool. (B) Comparison of Delta G (ΔG) values between *GATA2* WT vs. 5 synonymous mutations (syn Mut), 5 common synonymous single nucleotide polymorphisms (SNP) from gnomAD and 5 pathogenic non-synonymous *GATA2* mutations (path Mut). Standard one-way ANOVA test was applied to calculate p-values (mean ±SD).


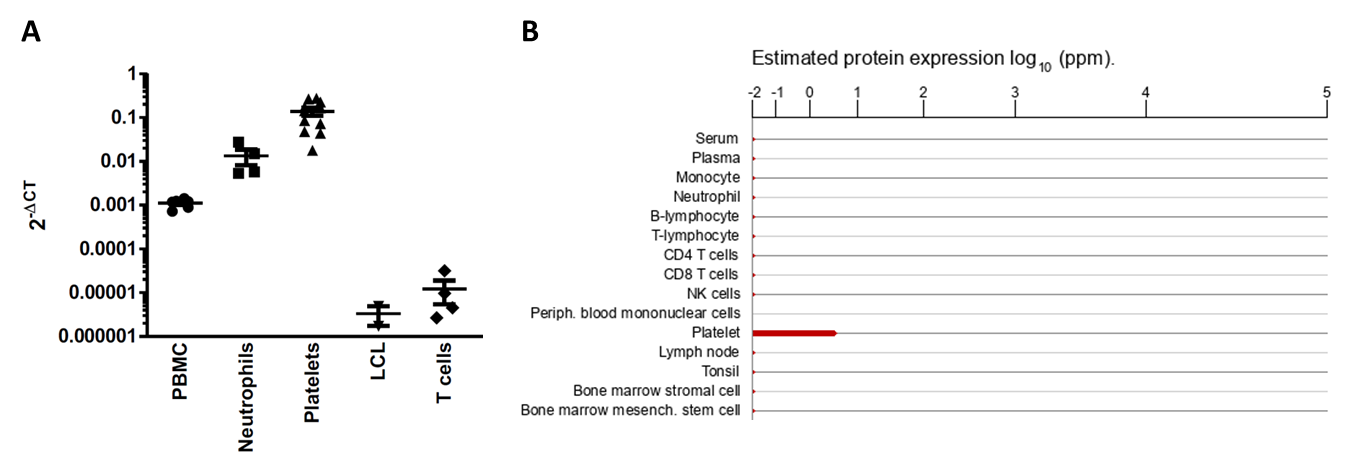


**Supplemental Figure 5 (linked to Figure 4). GATA2 expression and function in hematopoietic cells.** (A) The level of the *GATA2* gene expression was determined in various hematopoietic subpopulations of multiple healthy controls by RT-qPCR. (B) Graph adapted from the GeneCards database indicating the level of GATA2 protein in platelets (<https://www.genecards.org/cgi-bin/carddisp.pl?gene=GATA2#protein_expression>; last visit: 12.12.2019 at 12:52pm GMT+1).


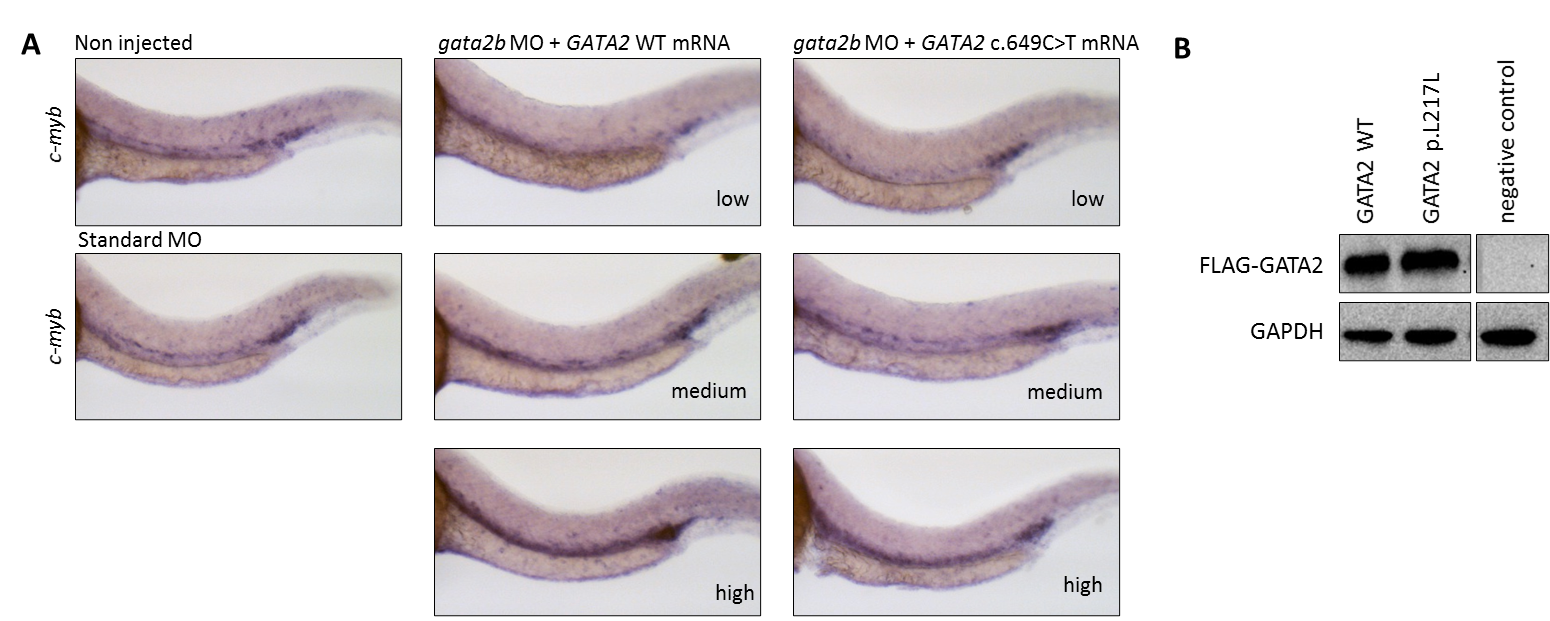


**Supplemental Figure 6 (linked to Figure 5). Analysis of zebrafish hematopoiesis *in vivo* and assay verification.** (A) Representative pictures of GATA2 WT/Mut-injected embryos manifesting with low, medium and high phenotype. (B) The level of human GATA2 protein expressed from the plasmid used for *FLAG-GATA2* mRNA generation for zebrafish injections. One representative blot is shown.

**Supplemental Table 1.** Overview of the variants identified using whole exome sequencing in patients with synonymous *GATA2* mutations.

| **Patient** | **Gene/ Isoform** | **Mutation** | **Type** | **VAF/**  **Total depth** | **gnomAD MAF% (ALT/total)** | **CADD/ GAVIN** | **Remarks** |
| --- | --- | --- | --- | --- | --- | --- | --- |
| P1 | *GATA2*  NM_032638.4 | c.351C>G  p.T177T | het | 50%/ 160 | none | 10.5/  B | Causes monoallelic *GATA2* RNA degradation (shown here) |
|  | *FANCD1 (BRCA2)*  NM_000059 | c.7863T>A  p.Y2621X | het | 44%/ 61 | none | 40/  LP, C4 | Biallelic mutations cause FA;  heterozygous VUS not considered disease-causing in this patient |
| P3 | *GATA2*  NM_032638.4 | c.351C>G  p.T177T | het | 47%/ 130 | none | 10.5/ B | Causes monoallelic *GATA2* RNA degradation (shown here) |
| P4 | *GATA2*  NM_032638.4 | c.649C>T  p.L217L | het | 48%/ 196 | 0.001%  (2/246096) | 10.7/ B | Causes partial *GATA2* RNA degradation (shown here) |
|  | *MAP2K1*  NM_002755 | c.65G>T  p.G22V | het | 45%/ 300 | none | 22.4/ LP, C1 | Monoallelic mutations in the MEK functional domain (starting at amino acid 62) cause Noonan/CFC syndrome; VUS found here is outside of a functional domain and not considered disease-causing in this patient |
| P5 | *GATA2*  NM_032638.4 | c.981G>A  p.G327G | het | 52%/ 156 | none | 18.5/ B | Causes monoallelic *GATA2* RNA degradation |
|  | *PIGT*  NM_015937 | c.514C>T  p.R172C | het | 47%/ 177 | 0.001% (3/245988) | 35/ LP, C4 | Monoallelic germline splice site mutation in *PIGT* has been reported in a single patient with PNH-2 disease presenting with severe hemolysis;  heterozygous VUS not considered disease-causing in this patient |
| P6 | *GATA2*  NM_032638.4 | c.1023C>T  p.A341A | het | 47%/ 296 | 0.002% (6/275438) | 15.4/ B | Causes monoallelic *GATA2* RNA degradation |
|  | *SAMD9*  NM_001193307 | c.1098delT  p.F366LfsX33 | het | 29%/ 52 | none | -/ - | Monoallelic mutations cause MIRAGE syndrome/familial MDS |
|  | *SAMD9*  NM_001193307 | c.2629A>G  p.K877E | het | 32%/ 41 | none | 7.6/ - | see above |
|  | *FANCD2*  NM_001018115 | c.3371G>A  p.S1124N | het | 57%/ 68 | none | 23.1/ LP, C4 | Biallelic mutations cause FA;  heterozygous VUS not considered disease-causing in this patient |
| P7 | *GATA2*  NM_032638.4 | c.1416G>A  p.P472P | het | 49%/ 63 | 0.027% (70/256322) | 12.4/ B | Causes monoallelic *GATA2* RNA degradation (shown here) |
|  | *FANCS (BRCA1)*  NM_007294 | c.2596C>T*  p.R866C | het | 51%/ 90 | 0.015%  (36/245842) | 33/ LP, C4 | Biallelic mutations cause FA;  heterozygous VUS not considered disease-causing in this patient |

All mutations are novel with the exception of FANCS (*) and some of the GATA2 variants. Abbreviations: VAF, variant allelic frequency; MAF, minor allele frequency; ALT, alternative allele; CADD, Combined annotation-dependent depletion score; GAVIN, Gene-Aware Variant Interpretation; B, benign; LP, likely pathogenic; FA, Fanconi anemia; VUS, variant of uncertain significance; MEK, mitogen-activated protein kinase kinase; CFC, Cardiofaciocutaneous syndrome; PNH-2, Paroxysmal nocturnal hemoglobinuria 2; MIRAGE, myelodysplasia, infection, restriction of growth, adrenal hypoplasia, genital phenotypes, and enteropathy. Variants with CADD-scores higher than the pathogenic threshold obtained from GAVIN were considered as likely pathogenic with the exception of *SAMD9* and synonymous *GATA2* mutations. GAVIN 5.0 thresholds: *GATA2* (pathogenic if CADD > 26.03, benign if < 19.23; C2), *FANCD1* (pathogenic if CADD > 32.6, benign if < 16.47; C4), *MAP2K1* (pathogenic if CADD > 23.5, benign if < 20.32; C1), *PIGT* (pathogenic if CADD > 36.6, benign if < 20.14; C4), *FANCD2* (pathogenic if CADD > 34, benign if < 10.23; C4), *FANCS* (pathogenic if CADD > 30, benign if < 13.06; C4). Explanation of GAVIN gene calibration categories: C1 = CADD scores highly significantly predictive for pathogenicity (*p* < 0.01); C2 = CADD scores significantly predictive for pathogenicity (*p* < 0.05); C3 = CADD scores may be predictive for pathogenicity (*p* > 0.05 but with few samples); C4 = CADD scores less predictive for pathogenicity (*p* > 0.05 with enough samples).

**Supplemental Table 2.** Sequence and position of *GATA2* specific primers used for cDNA sequencing. For primer design, binding sites were selected that do not contain polymorphic sequence (single nucleotide variation and copy number variation).

| **Mutation** | **Primer pair** | **Primer sequence** | **Start** | **End** |
| --- | --- | --- | --- | --- |
| c.351C>G (P1-3) | F1 | TACAGCCCCGCGCACGCCCG | c.214 | c.233 |
|  | R1 | CCTGGGTACACAGAGAGTG | c.428 | c.410 |
| c.649C>T | F2 | GTCTCCAGCCTCATCTTCCG | c.573 | c.592 |
|  | R2 | GAAGAGTCCGCTGCTGTAG | c.795 | c.777 |
| c.981G>A | F3 | AGGCTCGTTCCTGTTCAGAAG | c.854 | c.874 |
|  | R3 | AAGGTGGTGGTTGTCGTCTG | c.1076 | c.1057 |
| c.1023C>T | F4 | AGGCTCGTTCCTGTTCAGAAG | c.854 | c.874 |
|  | R4 | CTTCCTTCTTCATGGTCAGTG | c.1174 | c.1154 |
| c.1416G>A | F5 | GGAGAAGTCATCCCCCTTCAGTG | c.1266 | c.1288 |
|  | R5 | CTAGCCCATGGCGGTCACCATGC | c.1443 | c.1421 |
| c.490G>A | F1 | TACAGCCCCGCGCACGCCCG | c.214 | c.233 |
|  | R6 | CCCATAGTAGCTAGGCCTGG | c.710 | c.691 |
| c.351C>G (P9) | F6 | CAACCCCTACTATGCCAACC | c.168 | c.187 |
|  | R7 | GTCGTCTGACAATTTGCACAAC | c.1064 | c.1043 |

**REFERENCES**

1. Wehr C, Grotius K, Casadei S, Bleckmann D, Bode SFN, Frye BC, et al. A novel disease-causing synonymous exonic mutation in GATA2 affecting RNA splicing. *Blood.* 2018;132(11):1211-5.
2. Fox TA, Chakraverty R, Burns S et al. Successful outcome following allogeneic hematopoietic stem cell transplantation in adults with primary immunodeficiency. *Blood*. 2018;131 (8):917-931.
3. Wlodarski MW, Hirabayashi S, Pastor V, Stary J, Hasle H, Masetti R, *et al*. Prevalence, clinical characteristics, and prognosis of GATA2-related myelodysplastic syndromes in children and adolescents. *Blood.* 2016;127(11):1387-1397.
4. Wang K, Li M, Hakonarson H. ANNOVAR: functional annotation of genetic variants from high-throughput sequencing data. *Nucleic Acids Res*. 2010;38(16):e164.
5. Flex E, Niceta M, Cecchetti S, Thiffault I, Au MG, Capuano A, *et al*. Biallelic Mutations in TBCD, Encoding the Tubulin Folding Cofactor D, Perturb microtubule dynamics and cause early-onset encephalopathy. *Am J Hum Genet.* 2016;99(4):962-973.
6. Kortüm F, Caputo V, Bauer CK, Stella L, Ciolfi A, Alawi M, *et al*. Mutations in KCNH1 and ATP6V1B2 cause Zimmermann-Laband syndrome. *Nat Genet.* 2015;47(6):661-667.
7. Pastor VB, Sahoo SS, Boklan J, Schwabe GC, Saribeyoglu E, Strahm B, *et al*. Constitutional SAMD9L mutations cause familial myelodysplastic syndrome and transient monosomy 7. *Haematologica.* 2018;103(3):427-437.
8. Ma X, Liu Y, Liu Y, Alexandrov LB, Edmonson MN, Gawad C, *et al*. Pan-cancer genome and transcriptome analyses of 1,699 paediatric leukaemias and solid tumours. *Nature.* 2018; 555(7696):371-376.
9. Gröbner SN, Worst BC, Weischenfeldt J, Buchhalter I, Kleinheinz K, Rudneva VA, *et al.* The landscape of genomic alterations across childhood cancers. *Nature.* 2018;555(7696):321-327.
10. Lindsley RC, Saber W, Mar BG, Redd R, Wang T, Haagenson MD, *et al*. Prognostic Mutations in Myelodysplastic Syndrome after Stem-Cell Transplantation. *N Engl J Med.* 2017;376(6):536-547.
11. Schwartz JR, Ma J, Lamprecht T, Walsh M, Wang S, Bryant V, *et al*. The genomic landscape of pediatric myelodysplastic syndromes. *Nat Commun.* 2017;8(1):1557.
12. Papaemmanuil E, Gerstung M, Malcovati L, Tauro S, Gundem G, Van Loo P, *et al.* Clinical and biological implications of driver mutations in myelodysplastic syndromes. *Blood*. 2013; 122(22):3616-27.
13. Pastor V, Hirabayashi S, Karow A, Wehrle J, Kozyra EJ, Nienhold R, *et al.* Mutational landscape in children with myelodysplastic syndromes is distinct from adults: specific somatic drivers and novel germline variants. *Leukemia.* 2017;31(3):759-762.
14. Wlodarski MW, O'Keefe C, Howe EC, Risitano AM, Rodriguez A, Warshawsky I, et al. Pathologic clonal cytotoxic T-cell responses: nonrandom nature of the T-cell-receptor restriction in large granular lymphocyte leukemia. *Blood*. 2005;106(8):2769-80.
15. Zuker M. Mfold web server for nucleic acid folding and hybridization prediction. *Nucleic Acids Res.* 2003;31(13):3406-15.
16. Lorenz R, Bernhart SH, Honer Zu Siederdissen C, Tafer H, Flamm C, Stadler PF, et al. ViennaRNA Package 2.0. *Algorithms Mol Biol.* 2011;6:26.
17. Markham NR, and Zuker M. DINAMelt web server for nucleic acid melting prediction. *Nucleic Acids Res.* 2005;33(Web Server issue):W577-81.
18. Pertea M, Kim D, Pertea GM, Leek JT, and Salzberg SL. Transcript-level expression analysis of RNA-seq experiments with HISAT, StringTie and Ballgown. *Nat Protoc.* 2016;11(9):1650-67.
19. Desmet FO, Hamroun D, Lalande M, Collod-Beroud G, Claustres M, and Beroud C. Human Splicing Finder: an online bioinformatics tool to predict splicing signals. *Nucleic Acids Res.* 2009;37(9):e67.
20. Lek M, Karczewski KJ, Minikel EV, Samocha KE, Banks E, Fennell T, et al. Analysis of protein-coding genetic variation in 60,706 humans. *Nature.* 2016;536(7616):285-91.
21. Charan, J. & Kantharia, N. D. How to calculate sample size in animal studies? *J. Pharmacol. Pharmacother.* 2013; (4):303–306.
22. Butko E, Distel M, Pouget C, Weijts B, Kobayashi I, Ng K, et al. Gata2b is a restricted early regulator of hemogenic endothelium in the zebrafish embryo. *Development.* 2015;142(6):1050-61.
23. Thisse C, and Thisse B. High-resolution in situ hybridization to whole-mount zebrafish embryos. *Nat Protoc.* 2008;3(1):59-69.
24. Bolli N, Payne EM, Rhodes J, Gjini E, Johnston AB, Guo F, et al. cpsf1 is required for definitive HSC survival in zebrafish. *Blood.* 2011;117(15):3996-4007.
